# Leu-to-Phe substitution at prM^146^ decreases the growth ability of Zika virus and partially reduces its pathogenicity in mice

**DOI:** 10.1038/s41598-021-99086-2

**Published:** 2021-10-04

**Authors:** Takuya Inagaki, Satoshi Taniguchi, Yasuhiro Kawai, Takahiro Maeki, Eri Nakayama, Shigeru Tajima, Haruko Takeyama, Chang Kweng Lim, Masayuki Saijo

**Affiliations:** 1 Department of Virology I, National Institute of Infectious Diseases, Tokyo, Japan; 2 Department of Life Science and Medical Bioscience, Waseda University, Tokyo, Japan; 3 Division of Biosafety Control and Research, Management Department of Biosafety and Laboratory Animal, National Institute of Infectious Diseases, Tokyo, Japan

**Keywords:** Virology, Viral pathogenesis, Viral genetics, Pathogens

## Abstract

Zika virus (ZIKV) is a mosquito-borne flavivirus that causes febrile illness. The recent spread of ZIKV from Asia to the Americas via the Pacific region has revealed unprecedented features of ZIKV, including transplacental congenital infection causing microcephaly. Amino acid changes have been hypothesized to underlie the spread and novel features of American ZIKV strains; however, the relationship between genetic changes and the epidemic remains controversial. A comparison of the characteristics of a Southeast Asian strain (NIID123) and an American strain (PRVABC59) revealed that the latter had a higher replication ability in cultured cells and higher virulence in mice. In this study, we aimed to identify the genetic region of ZIKV responsible for these different characteristics using reverse genetics. A chimeric NIID123 strain in which the E protein was replaced with that of PRVABC59 showed a lower growth ability than the recombinant wild-type strain. Adaptation of the chimeric NIID123 to Vero cells induced a Phe-to-Leu amino acid substitution at position 146 of the prM protein; PRVABC59 also has Leu at this position. Leu at this position was found to be responsible for the viral replication ability and partially, for the pathogenicity in mouse testes.

## Introduction

Zika virus (ZIKV) is a member of the genus *Flavivirus*, which includes yellow fever virus, dengue virus, West Nile virus, and Japanese encephalitis virus^1–3^. Viruses in this group are typically transmitted by arthropod vectors and cause febrile and/or neurological diseases^1–3^. ZIKV was first isolated from a rhesus monkey in Uganda in 1947 and was endemic to African and Asian countries until 2007^3,4^. The first outbreak of ZIKV infections in humans (ZIKV diseases, ZVD) outside Africa and Asia occurred in the Pacific islands in 2007^5^. Since then, ZIKV has spread via the Pacific islands^6,7^ to the Americas, where it caused a large epidemic in 2015 and 2016^3,8,9^. ZIKV is transmitted by *Aedes* mosquitos and causes a febrile disease with symptoms including rash, headache, joint and muscle pain, and conjunctivitis^3^. The epidemic in the Pacific and Americas revealed unprecedented properties of ZVD. ZIKV can infect fetuses through the placenta, resulting in congenital ZVD with neurological disorders, such as microcephaly, and abortion^3,10–12^. ZIKV infection also causes neurological complications, such as Guillain-Barré syndrome^13^. ZIKV infects diverse cell types and organs, including the testes^14^; ZIKV has been detected in seminal specimens and infectious ZIKV has been isolated from semen^15–17^. Cases of ZIKV transmission via sexual intercourse have also been documented^17–20^.

ZIKV has a single-stranded, positive-sense RNA genome that encodes three structural proteins (C, prM, and E) and seven nonstructural proteins (NS1, NS2A, NS2B, NS3, NS4A, NS4B, and NS5) from a single open reading frame, and noncoding regions in its 5′- and 3′-termini^1^. Some evidence suggests that the accumulation of amino acid changes that increase virus infectivity and/or virulence underlaid the epidemic and the discovery of the unprecedented features of ZVD^21–24^. ZIKV is geographically and genetically divided into African and Asian/American lineages, and those are the Asian lineages that have spread to the Americas through the Pacific^3,24,25^. During the spread of the virus to the Pacific and Americas, the ZIKV genome accumulated amino acid changes^3,24,26^. Several amino acid substitutions in the ZIKV polyproteins have been experimentally demonstrated to enhance the infectivity and/or virulence in mice^21–24^. However, microcephaly cases with autochthonous ZIKV infection have also been reported in Asian countries^27–29^. In addition, African ZIKV strains cause fetal damage in mice^30,31^. A report suggests that an amino acid substitution that emerged after the ZVD epidemic in Brazil does not affect the replication and virulence of ZIKV^26,32^. Thus, the relationship between genetic variation in ZIKV and its replication capacity and pathogenicity remains controversial.

ZIKV Asian/American lineages can be subdivided into Southeast Asian, Pacific, and American subtypes, which have different amino acid sequences^33,34^. Previously, we compared ZIKV strains of the three subtypes, i.e., the Southeast Asian subtype ZIKV/Hu/NIID123/2016 (NIID123), the Pacific subtype ZIKV/Hu/Chiba/S36/2016, and the American subtype PRVABC59, in terms of their replication capacity in cell culture and virulence in interferon-alpha/beta receptor knock-out (IFNAR1-KO) mice^34^. PRVABC59 showed the highest replication potential in vitro and in mice, while NIID123 had the lowest replication ability in vitro and in vivo^34^. In addition, mice inoculated with PRVABC59 often showed testicular atrophy and scarring, whereas no testis damage was seen in mice inoculated with NIID123 at 6 weeks post infection^34^. These findings indicated that genetic differences between PRVABC59 and NIID123 are involved in the replication ability and pathogenicity of the viruses.

In the present study, we sought to identify the region responsible for the replication ability and pathogenicity of ZIKV strains using various recombinant ZIKV. To generate recombinant chimeric and point mutant ZIKV, we first developed a novel reverse genetics system for the PRVABC59 and NIID123 strains based on the infectious subgenomic amplicons (ISA) method^35,36^. The properties of the recombinant ZIKV produced using the system were evaluated in cultured cells and mice. Our previous study showed that IFNAR1-KO mice infected with PRVABC59- or NIID123 are not lethal^34^. Therefore, the level of testis damage in ZIKV-infected mice was used as an index for assessing ZIKV pathogenicity in this study.

## Results

### Development of a reverse genetics system for the ZIKV NIID123 and PRVABC59 strains

**Figure 1:**
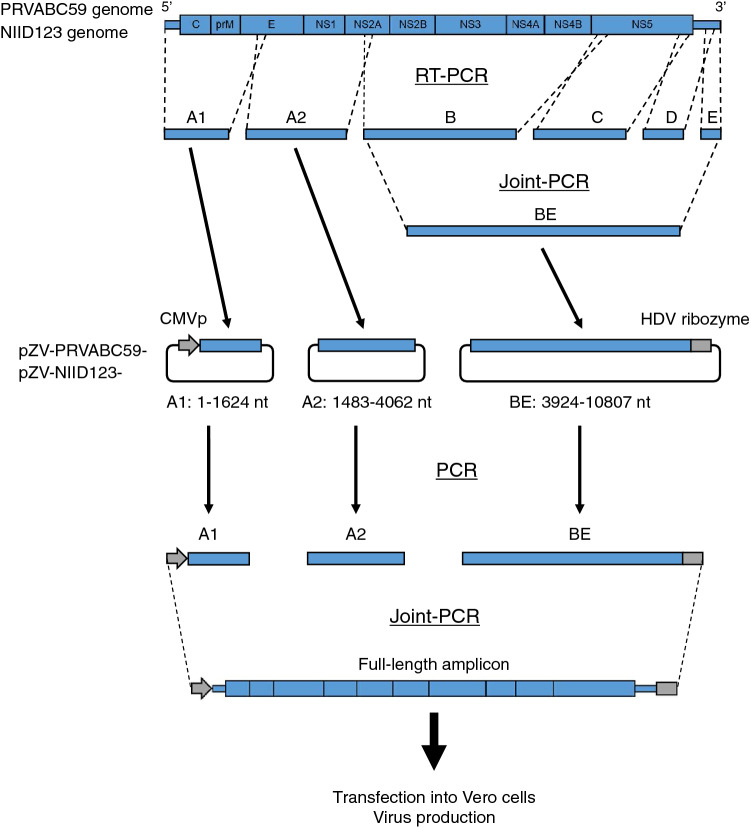
Schematic diagram of the reverse genetics system used for the ZIKV PRVABC59 and ZIKV/Hu/NIID123/2016 (NIID123) strains. For cloning, the whole-genome cDNA of ZIKV was divided into six regions (A1, A2, B, C, D, and E). pMW119 and pMW119-CMVP, which has CMV promoter and HDV ribozyme sequences in the multicloning site of pMW119, were used^37,38^. Fragments A1 and A2 were cloned into pMW119-CMVP and pMW119, respectively. Fragments B, C, D, and E were concatenated by joint PCR and then cloned into pMW119-CMVP. Six plasmid clones, pZV-PRVABC59-A1, pZV-PRVABC59-A2, pZV-PRVABC59-BE, pZV-NIID123-A1, pZV-NIID123-A2, and pZV-NIID123-BE, were obtained. To construct the full-length virus cDNA, the fragments in the clones were PCR-amplified and concatenated as full-length viral cDNA by joint PCR. The full-length viral cDNA with the CMV promoter sequence in the 5′-terminus and the HDV ribozyme sequence in the 3′-terminus was transfected into Vero cells to produce infectious viruses.

Two recombinant ZIKV, rPRVABC59 and rNIID123, were generated (Fig. 1). The nucleotide sequence of rNIID123 was identical to that of the parent virus NIID123. While the parental PRVABC59 had mixed nucleotides at positions 1965 (G and U) and 2781 (U and G), rPRVABC59 had U at both positions (Table 1).

**Table 1:** Summary of the genome sequences of the viruses used in this study.

PRVABC59 Nucleotide (amino acid)	Reference (KU501215)	paPRVABC59	rPRVABC59	rPRVABC59 prM^L146F^	
911 (268)	G (Leu)	G (Leu)	G (Leu)	U (Phe)	
1965 (620)	G (Val)	G/U (Val/Leu)	U (Leu)	U (Leu)	
2781 (892)	U (Trp)	U/G (Trp/Gly)	U (Trp)	U (Trp)	
10,749–10,807	Unavailable	Not determined	Extrapolated*	Extrapolated*	

NIID123 Nucleotide (amino acid)	Reference (LC219720)	paNIID123	rNIID123	rNIID123 E^PRV^	rNIID123 prM^F146L^
911 (268)	U (Phe)	U (Phe)	U (Phe)	U (Phe)	G (Leu)
1228 (374)	G (Arg)	G (Arg)	G (Arg)	A (Lys)	G (Arg)
1756 (550)	A (Asn)	A (Asn)	A (Asn)	G (Ser)	A (Asn)
1965 (620)	G (Val)	G (Val)	G (Val)	U (Leu)	G (Val)
2786 (893)	A (Arg)	U (Ser)	U (Ser)	U (Ser)	U (Ser)
10,749–10,807	Unavailable	Not determined	Extrapolated*	Extrapolated*	Extrapolated*

*pa* parent, *r* recombinant.

*The nucleotide sequence was extrapolated from that deposited in GenBank (MH158237).

**Figure 2:**
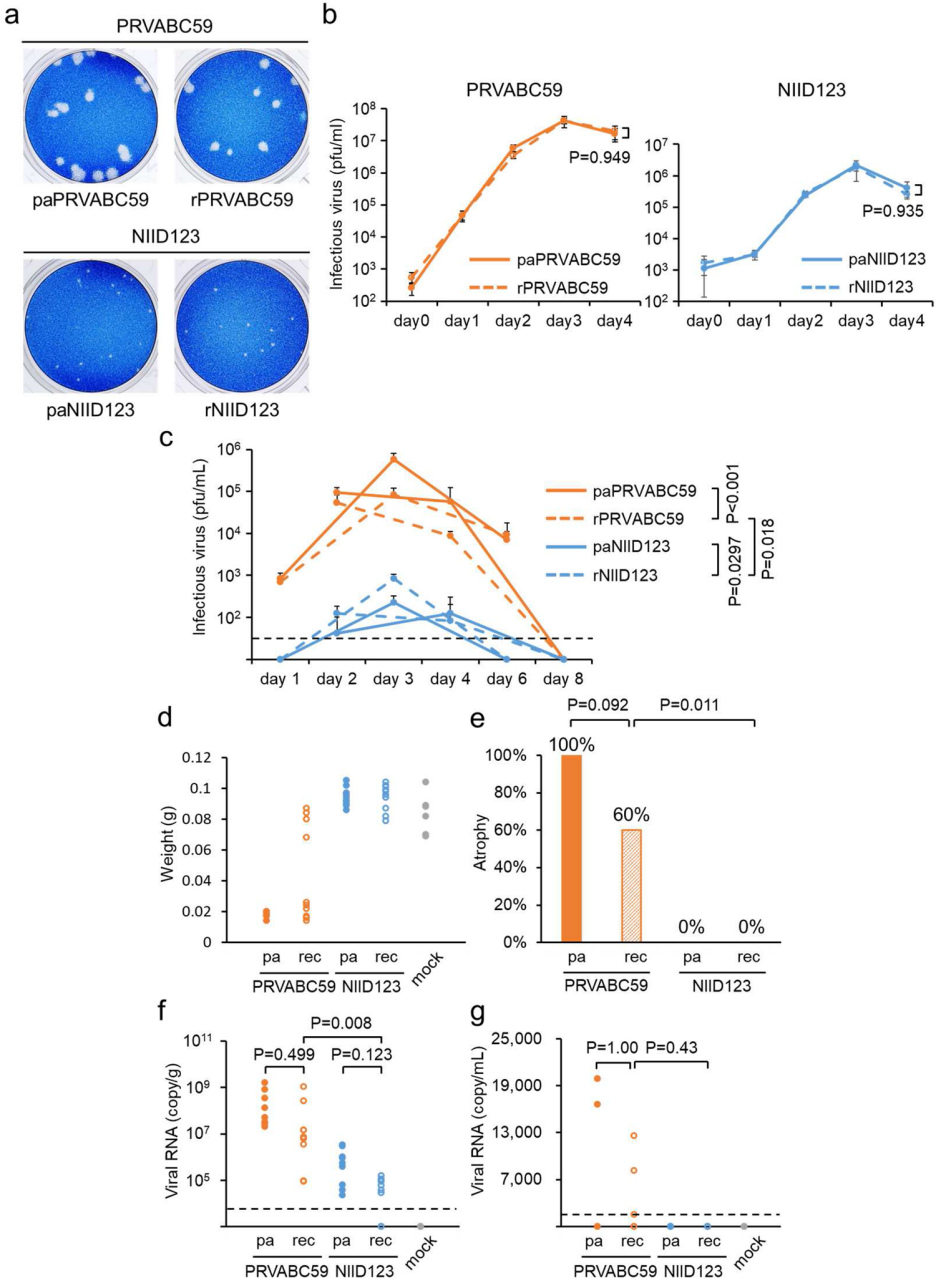
Characterization of recombinant ZIKV rPRVABC59 and rNIID123 in vitro and in vivo. (**a**) Plaque morphology of the parental virus PRVABC59 (paPRVABC59) and rPRVABC59 (left), and the parental virus NIID123 (paNIID123) and rNIID123 (right) in Vero cells at 5 days post infection. (**b**) Growth curves of parental and recombinant PRVABC59 (left), and parental and recombinant NIID123 (right) in Vero cells. Cells were inoculated with the viruses at an MOI of 0.01 pfu/cell. Means ± standard deviations of three independent experiments are shown. (**c**) Viremia levels in mice inoculated with parental and recombinant PRVABC59, and parental and recombinant NIID123. IFNAR1-KO mice (4 or 5 mice/virus group) inoculated with parental or recombinant virus via the footpad were divided into two subgroups (2 or 3 mice/subgroup; 1-, 3-, and 6-days post-infection group and 2-, 4-, and 8-day post-infection group) and blood was collected at the indicated time points. Means + standard deviations are shown. (**d**) Testis weights at 6 weeks post infection. (**e**) Ratios of atrophied to total testes in mice infected with the indicated viruses. (**f**,**g**) Viral RNA copy numbers in testes (**f**) and serum (**g**) at 6 weeks post infection. Dotted line: limit of detection. pa: parental virus. rec: recombinant virus.

The sizes of the plaques formed in Vero cells by the recombinant viruses were equivalent to those formed by the parental viruses (Fig. 2a). In addition, the growth kinetics of the recombinant viruses were nearly identical to those of the parental viruses (Fig. 2b).

The viremia levels in IFNAR1-KO mice infected with rPRVABC59 were similar to those infected with PRVABC59 (Fig. 2c). Similarly, the viremia levels in mice infected with rNIID123 were nearly the same as those in NIID123-infected mice (Fig. 2c). The levels in parental or rPRVABC59-infected groups were higher than those in parental or rNIID123-infected groups (Fig. 2c). Testis weight was decreased in all mice infected with PRVABC59 and in 60% of the testes of mice infected with rPRVABC59 at 6 weeks after inoculation (Fig. 2d,e). No testicular atrophy was observed in mice infected with parental or recombinant NIID123 (Fig. 2d,e). Viral RNA levels in rPRVABC59-infected mouse testes were higher than those in rNIID123-infected mouse testes (Fig. 2f). Viral RNA was also detected in several serum samples of parental or recombinant PRVABC59-infected mice at 6 weeks after inoculation (Fig. 2g). These data indicated that the recombinant viruses largely maintain the growth properties of the parental viruses both in vitro and in vivo.

### Replication capacity of an NIID123 strain in which the E protein was replaced with that of PRVABC59

**Figure 3:**
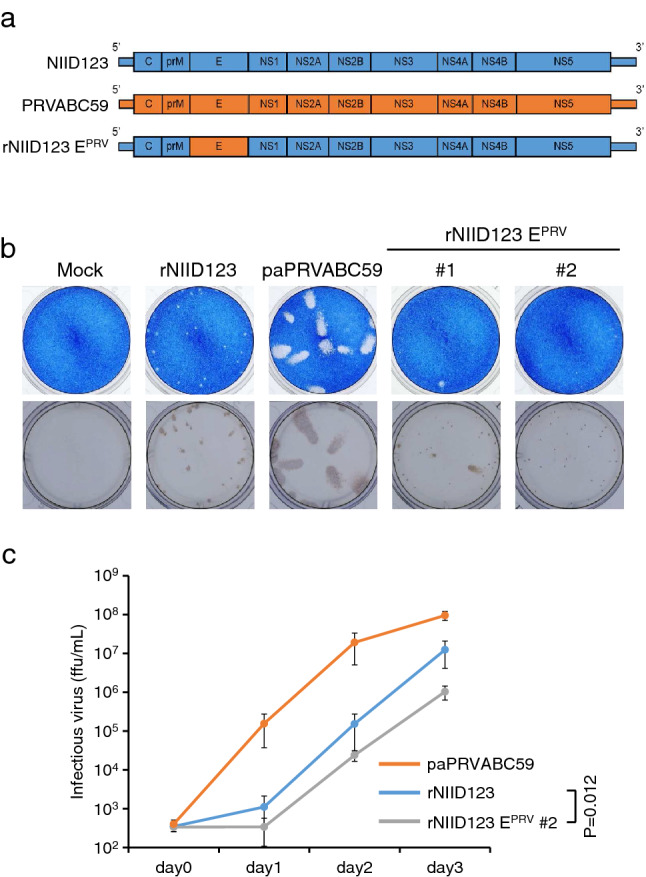
Generation and characterization of chimera ZIKV rNIID123 E^PRV^ in vitro. (**a**) Schematic representation of the genome structure of rNIID123 E^PRV^. (**b**) Plaques (upper panels) and focus phenotypes (lower panels) of rNIID123, paPRVABC59, and rNIID123 E^PRV^ #1 and #2 in Vero cells. (**c**) Growth curves for the parental virus paPRVABC59, rNIID123, and rNIID123 E^PRV^ #2. Cells were inoculated with the viruses at an MOI of 0.01 ffu/cell. Means ± standard deviations of three independent experiments are shown.

ZIKV E protein plays a crucial role in the virus replication cycle^39–41^, suggesting that it may be associated with the differences in growth ability and pathogenicity between the PRVABC59 and NIID123 strains. A recombinant chimeric ZIKV rNIID123 E^PRV^, in which the E region of NIID123 was replaced with that of rPRVABC59, was generated (Fig. 3a). rNIID123 E^PRV^ was produced twice independently and two culture lots of rNIID123 E^PRV^ (#1 and #2) were obtained (Fig. 3b). Both lots of rNIID123 E^PRV^ formed pinhole plaques, which were smaller than those produced by NIID123, on Vero cells at 5 days post-infection (Fig. 3b, upper panel). The plaques formed by rNIID123 E^PRV^ were confirmed by a focus assay (Fig. 3b, lower panel). rNIID123 E^PRV^ #2 was used for in vitro growth analysis because the viral titer of rNIID123 E^PRV^ #1 was very low, and the plaque and focus morphologies of lot #1 were heterogenous, existing with a few large plaque variants (Fig. 3b). rNIID123 E^PRV^ #2 grew significantly slower than rNIID123 in Vero cells (Fig. 3c).

### Effect of phenylalanine-to-leucine (Phe-to-Leu) substitution at position 146 of the prM protein in the adaptation of rNIID123 E^PRV^ to Vero cells

**Figure 4:**
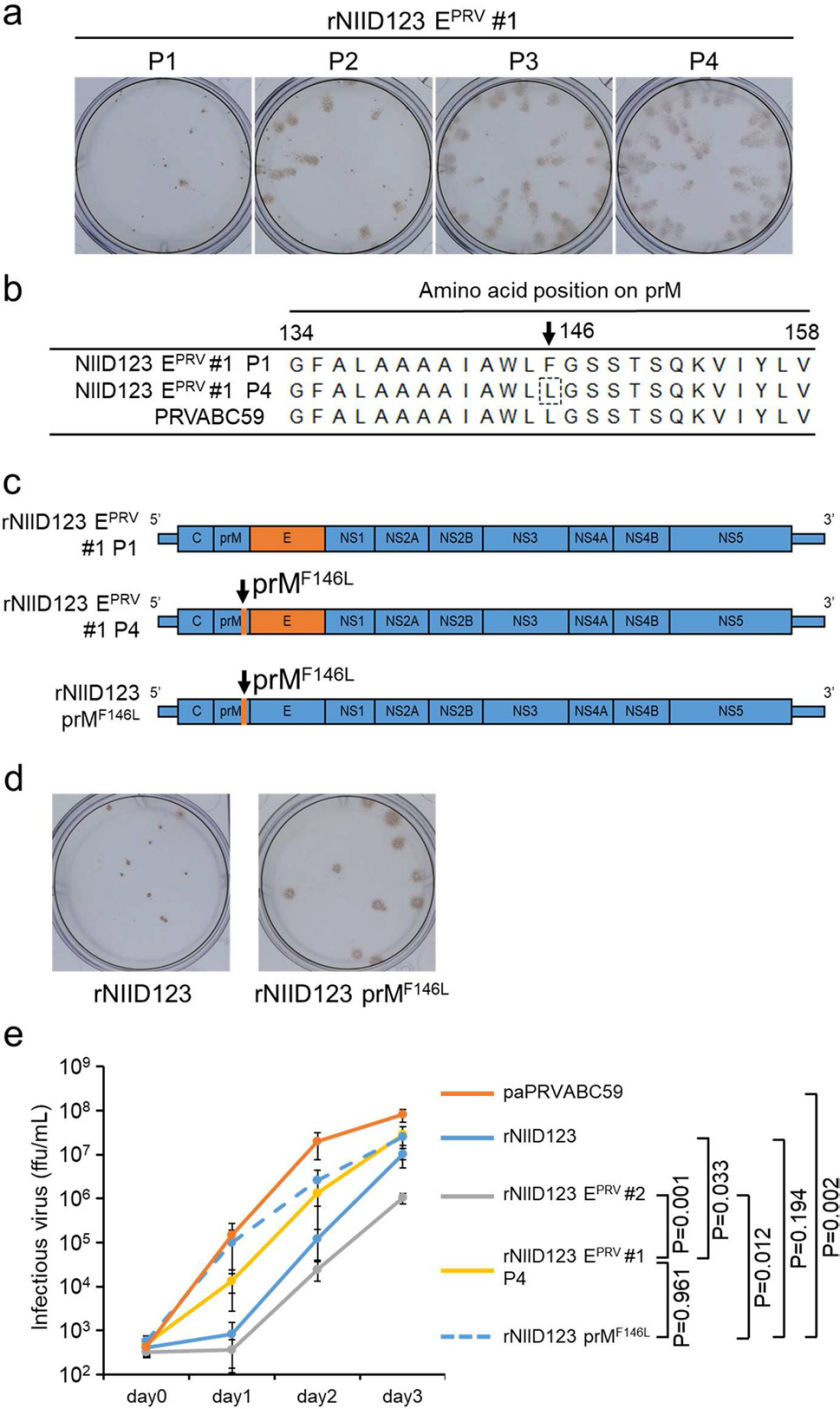
Recovery of Vero cell-adapted rNIID123 E^PRV^ and in vitro growth of the point mutant rNIID123 prM^F146L^. (**a**) Focus phenotypes of rNIID123 E^PRV^ #1 after one (P1) to four (P4) passages in Vero cells. (**b**) Partial amino acid sequences of the prM region in rNIID123 E^PRV^ #1 P1, P4, and PRVABC59. Numbers indicate positions in the ZIKV prM protein. (**c**) Schematic representation of the point mutant rNIID123 prM^F146L^. (**d**) Focus phenotypes of rNIID123 and rNIID123 prM^F146L^ in Vero cells. (**e**) Growth curves of rNIID123 E^PRV^ P4 and rNIID123 prM^F146L^ in Vero cells. Cells were inoculated with the viruses at an MOI of 0.01 ffu/cell. Means ± standard deviations of three independent experiments are shown.

To understand the characteristics underlying the decrease in rNIID123 E^PRV^ replication capacity, rNIID123 E^PRV^ #1 existing with the large plaque-variants was serially passaged in Vero cells. The Vero cell-adapted variants rNIID123 E^PRV^ #1 were obtained after four passages (rNIID123 E^PRV^ #1 P4), forming significantly larger foci than the original rNIID123 E^PRV^ (Fig. 4a, Supplementary Fig. S1). Whole-genome sequencing showed that rNIID123 E^PRV^ P4 had only one amino acid substitution (Phe to Leu) at position 146 of the prM protein (F146L) (corresponding to position 268 of the ZIKV polyprotein) (Fig. 4b). PRVABC59 also has Leu at this position (Fig. 4b). Next, a Phe-to-Leu substitution at position 146 of prM was introduced in NIID123 (rNIID123 prM^F146L^) to evaluate the substitution effect on the growth of NIID123 (Fig. 4c). Foci formed by rNIID123 prM^F146L^ were larger than those formed by rNIID123 in Vero cells (Fig. 4d, Supplementary Fig. S1). The growth rate of rNIID123 prM^F146L^ was similar to that of rNIID123 E^PRV^ P4 and higher than those of rNIID123 and rNIID123 E^PRV^ #2, although PRVABC59 showed a higher growth ability than rNIID123 prM^F146L^ (Fig. 4e).

### Effect of Leu-to-Phe substitution at position 146 of the prM protein on the growth ability of PRVABC59 in vitro

**Figure 5:**
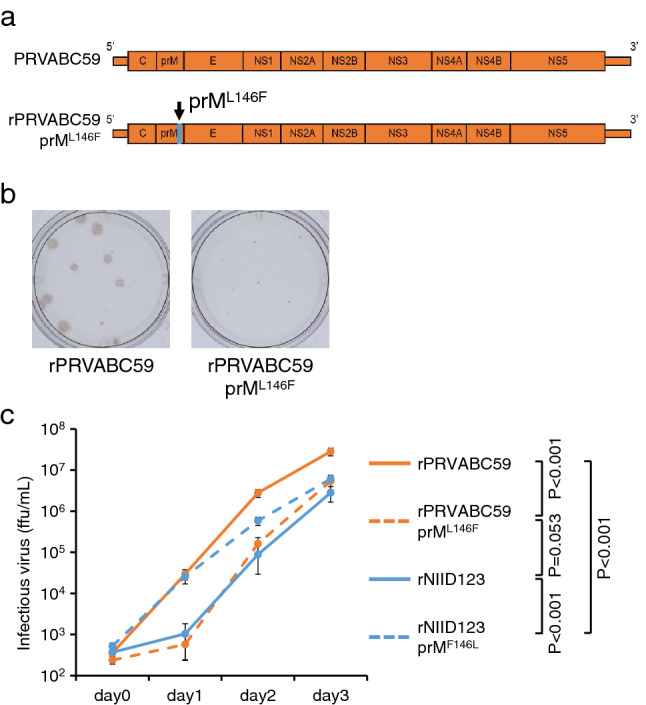
In vitro growth of the point mutant rPRVABC59 prM^L146F^. (**a**) Schematic representation of the mutant rPRVABC59 prM^L146F^. (**b**) Focus phenotypes of rPRVABC59 and rPRVABC59 prM^L146F^ in Vero cells. (**c**) Growth curves of the point mutants rPRVABC59 prM^L146F^ and rNIID123 prM^F146L^ in Vero cells. Cells were inoculated with the viruses at an MOI of 0.01 ffu/cell. Means ± standard deviations of three independent experiments are shown.

To evaluate the role of the amino acid residue at position 146 of prM in the in vitro growth ability of ZIKV further, PRVABC59 with a Leu-to-Phe substitution at this position of prM was generated (rPRVABC59 prM^L146F^) (Fig. 5a). rPRVABC59 prM^L146F^ formed markedly smaller foci than rPRVABC59 (Fig. 5b, Supplementary Fig. S1). The growth kinetics of rPRVABC59 prM^L146F^ were lower than those of rPRVABC59 and rNIID123 prM^F146L^, but similar to that of rNIID123 (Fig. 5c).

### Effect of amino acid substitution at position 146 of the prM protein of ZIKV PRVABC59 and NIID123 strains on viremia/viral RNA levels and testis damage in mice

**Figure 6:**
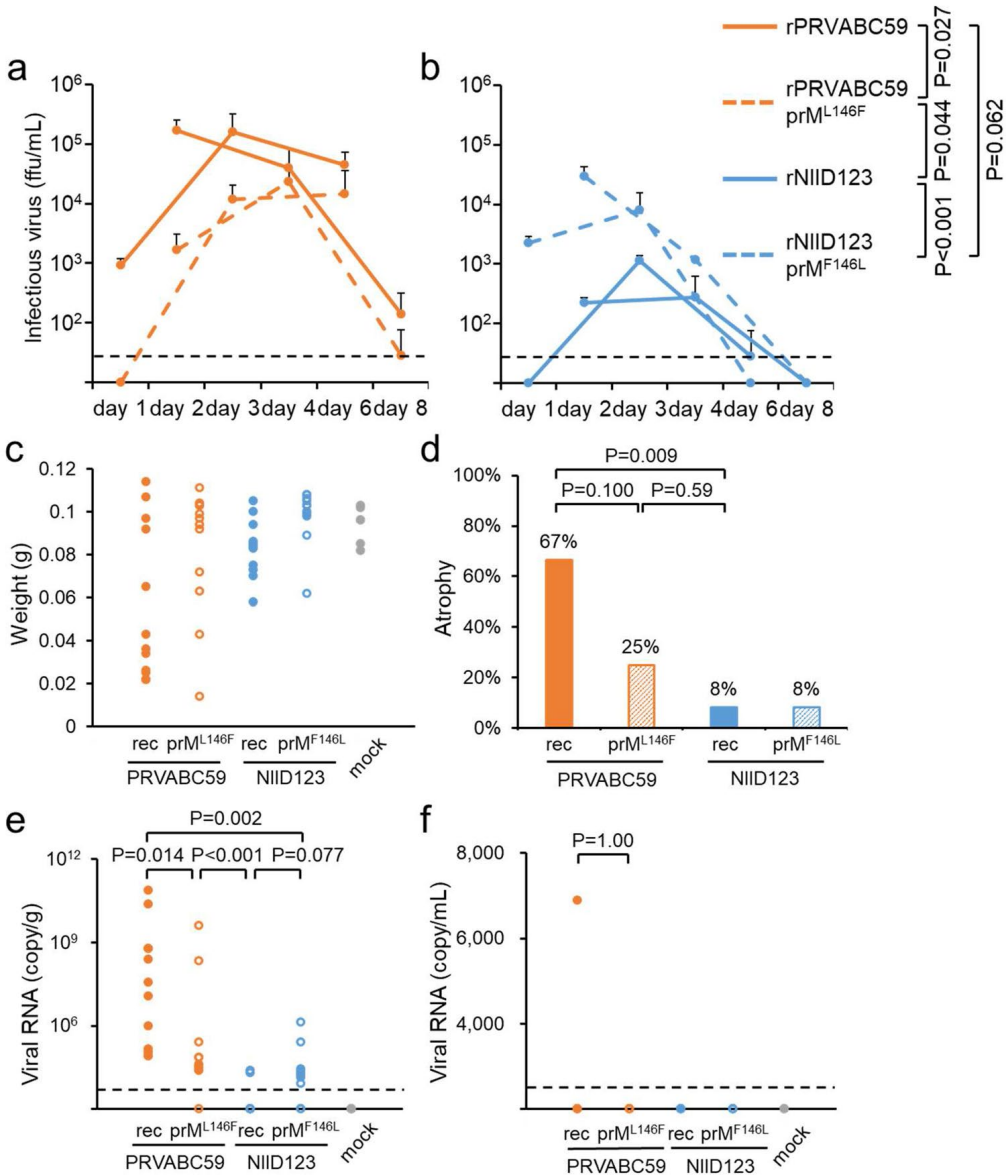
Characterization of the point mutants rPRVABC59 prM^L146F^ and rNIID123 prM^F146L^ in mice. (**a,b**) IFNAR1-KO mice (6 mice/virus group) inoculated with rPRVABC59,rPRVABC59 prM^L146F^, rNIID123 and rNIID123 prM^F146L^ via the footpad were divided into two subgroups (3 mice/subgroup; 1-, 3-, and 6-days post-infection group and 2-, 4-, and 8-days post-infection group) and blood was collected at the indicated time points. Means + standard deviations are shown. (**a**) rPRVABC59 and rPRVABC59 prM^L146F^. (**b**) rNIID123 and rNIID123 prM^F146L^. (**c**) Testis weight at 6 weeks post infection. (**d**) Ratios of atrophied to total testes in mice infected with the indicated viruses. (**e**,**f**) Viral RNA copy numbers in testes (**e**) and serum (**f**) at 6 weeks post infection. Dotted line: limit of detection.

Mice inoculated with rPRVABC59 prM^L146F^ showed lower viremia levels than those inoculated with rPRVABC59, while mice inoculated with rNIID123 prM^F146L^ showed higher viremia levels than those inoculated with rNIID123 (Fig. 6a,b). At 6 weeks post inoculation, 67% of the testes of rPRVABC59-infected mice were atrophied, whereas only 25% of the testes of rPRVABC59 prM^L146F^-infected mice showed atrophy (Fig. 6c,d). While testis weight was decreased in mice infected with rNIID123 and rNIID123 prM^F146L^, the rates of testis damage in both groups were lower than those in rPRVABC59-infected groups (8%) (Fig. 6c,d). Viral RNA levels in the testes of rPRVABC59 prM^L146F^-infected mice were lower than those in the testes of rPRVABC59-infected mice, but no noticeable difference was found between rNIID123- and rNIID123 prM^F146L^-infected mice (Fig. 6e). Viral RNA in the serum was detected in only one of the mice infected with rPRVABC59 at 6 weeks after inoculation (Fig. 6f).

## Discussion

Based on their amino acid sequences, Asian/American-lineage ZIKV strains are classified into Southeast Asian, Pacific, and American subtypes^33,34^. Our previous study demonstrated that the American subtype ZIKV strain PRVABC59 has a higher growth ability in cell culture and a higher pathogenicity in mice than the Southeast Asian subtype strain NIID123^34^. In this study, we developed a reverse genetics system for the PRVABC59 and NIID123 strains. We aimed to determine the genetic regions involved in the in vitro and in vivo phenotypic differences between the strains. Our findings indicated that the amino acid at position 146 of the prM protein is responsible for the different properties in vitro and partially for those in mice.

We first established a reverse genetics system to produce recombinant mutant PRVABC59 and NIID123 strains. We previously established a reverse genetics system for an African lineage ZIKV strain, MR766, by constructing a full-length cDNA clone of the strain using pMW119-CMVP plasmid^37^. In this study, we first attempted to construct complete cDNA clones of PRVABC59 and NIID123 strains. The PRVABC59 and NIID123 genomes could not be maintained or stably amplified in the plasmid and competent *Escherichia coli* host cells. Therefore, we used the ISA method, with some modifications, to produce recombinant viruses (Fig. 1). In the modified method, genomic cDNA fragments of the viruses were combined by joint PCR to form a complete genomic cDNA of the viruses with a CMV promoter and HDV ribozyme sequences. Using this system, we were able to prepare infectious recombinant ZIKV without constructing a full-length genomic cDNA clone. Therefore, this reverse genetics system may be more stable and more convenient for the production of recombinant viruses of not only other ZIKV strains, but also other flaviviruses, as previously reported^35,36^.

The recombinant viruses rPRVABC59 and rNIID123 showed growth properties similar to those of the parental viruses in vitro (Fig. 2). However, the rate of testis damage in rPRVABC59-inoculated mice was slightly lower than that in parental PRVABC59-inoculated mice (Fig. 2). Parental PRVABC59, which was a clinically isolated virus, was a mixture of genetic variants (Table 1)^25,42^. In a previous study, ZIKV variants in the wild-type strain population showed different growth abilities in vitro and pathogenicity in interferon receptor KO mice^42^. rPRVABC59 population is predicted to have more homogeneity than parental PRVABC59 population, which may explain the phenotypic differences between the parental and recombinant PRVABC59 strains, as described in previous study^43^.

Flavivirus E protein is mainly involved in viral entry into cells and virion assembly and, thus, in viral growth ability and pathogenicity^39–41^. Therefore, we hypothesized that the growth ability of NIID123 would increase by replacing its E protein with that of PRVABC59, and generated the mutant ZIKV rNIID123 E^PRV^ (Fig. 3). However, the growth ability of rNIID123 E^PRV^ was lower than that of the parental virus rNIID123, indicating that E protein of PRVABC59 does not increase the low growth ability of NIID123 in vitro. rNIID123 E^PRV^ was passaged in Vero cells four times to obtain a Vero cell-adapted mutant virus with recovered growth capacity (Fig. 4). rNIID123 E^PRV^ P4 had only one amino acid substitution at position 146 of the prM protein (prM^146^): a Phe-to-Leu substitution. PRVABC59 also has Leu at prM^146^. Previous reports have indicated that ZIKV E and prM proteins form heterodimers during virion assembly^39,40^. The interaction with the E protein is predicted to promote the Phe-to-Leu substitution at prM^146^. However, the growth ability of rNIID123 prM^F146L^ in Vero cells was also increased (Fig. 4), indicating that the interaction of PRVABC59 E protein with the Leu residue at prM^146^ was not associated with the adaptation of rNIID123 E^PRV^ in Vero cells. The amino acid differences on prM and E proteins between PRVABC59 and NIID123 strains were summarized in Supplementary Table S1.

Phe-to-Leu substitution at prM^146^ of rNIID123 prM increased the growth ability of NIID123 in vitro and in vivo, but did not increase the rate of testis damage compared to that induced by rNIID123 (Figs. 5 and 6). In contrast, Leu-to-Phe substitution at the same site in rPRVABC59 did decrease the growth ability of rPRVABC59. Moreover, the pathogenicity of rPRVABC59 prM^L146F^ in mouse testes was lower than that of rPRVABC59, but higher than that of rNIID123 and rNIID123 prM^F146L^ (Figs. 5 and 6). These results suggest that the growth ability of ZIKV in vitro and in vivo does not necessarily reflect the virulence of the virus in mice.

Previous studies indicated that ZIKV prM protein is involved in growth capacity and cytotoxicity in cell culture and neurovirulence in mice^21,37,44–47^. Our study indicated that prM^146^ is associated with viral growth in Vero cells and is partially responsible for pathogenicity in mouse testes. prM^146^ is located in the transmembrane domain of the prM protein and near the interval of two α-helices and the inside of the viral envelope^48–51^. Structural studies of the protein have suggested that this portion serves as the interface for interaction with viral capsid protein, and this interaction may be associated with virion assembly of ZIKV^49,50^. Another structural study suggested that the transmembrane helices of the prM and E proteins form lipid-binding pockets and the interaction of the proteins with lipids is critical for the life cycle of ZIKV^51^. The amino acid residue at prM^146^ is not located in the interface nor in the pocket, but nearby them^50,51^. Therefore, the residue at this position may be associated with virion assembly.

**Figure 7:**
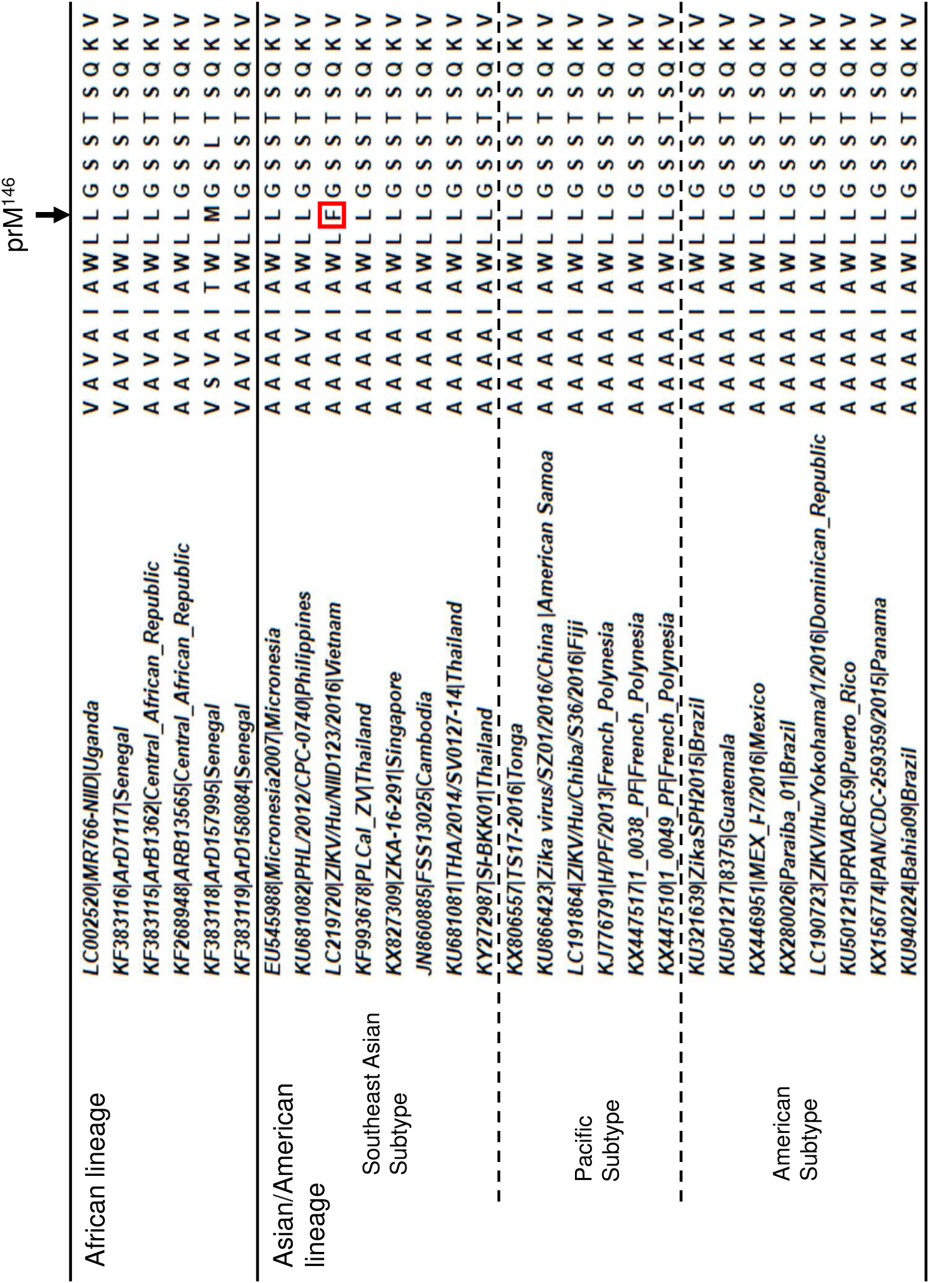
Alignment of partial amino acid sequences of the prM protein of ZIKV strains. Twenty-eight strains representing the African lineage and the Southeast Asian, Pacific, and American subtypes of the Asian/American lineage of ZIKV were used. The amino acid residue at position 146 of the prM protein in the NIID123 strain is indicated by a red rectangle.

As shown in Fig. 7, the Phe residue at prM^146^ is rarely seen in ZIKV isolates (Fig. 7), suggesting that the lower growth ability and virulence of NIID123 in vitro and in vivo may be a unique phenotype among ZIKV isolates. Further studies are needed to elucidate the role of prM^146^ in the replication ability and pathogenicity of ZIKV and to understand the nature of the ZIKV isolates.

## Materials and methods

### Ethics statement

All animal experiments in this study were conducted with approval (Nos. 120011 and 120,113) from the Animal Care and Use Committee of the National Institute of Infectious Diseases (NIID) and in compliance with the animal husbandry and welfare regulations of Japan, and the ARRIVE (Animal Research: Reporting of In Vivo Experiments) guidelines. Mouse experiments were performed in biosafety level 2 animal facilities at the NIID. For the collection of testis samples, mice were euthanized using isoflurane. All efforts were made to minimize any potential pain and distress.

### Cells and viruses

Vero9013 cells were cultured in Eagle’s minimal essential medium (MEM, Sigma-Aldrich, St. Louis, MO) supplemented with 10% fetal bovine serum (FBS, Sigma-Aldrich), 100 U/mL of penicillin, and 100 µg/mL of streptomycin (Nacalai Tesque, Kyoto, Japan) in an atmosphere of 5% CO_2_ at 37 °C. ZIKV strain PRVABC59 (GenBank accession No.: KU501215) was propagated in Vero cells for five passages and ZIKV strain ZIKV/Hu/NIID123/2016 (NIID123) was propagated once in mosquito C6/36 cells and then eight times in Vero cells, and culture supernatants were used as original virus solutions.

### Establishment of a reverse genetics system for the ZIKV PRVABC59 and NIID123 strains

We established a reverse genetics system for the strains using the ISA method^35,36^, with modification (Fig. 1). To determine the genome sequences of parental viruses, viral RNA was extracted from the original virus solutions using High Pure Viral RNA kit (Roche Diagnostics, Mannheim, Germany). Viral cDNA was synthesized with SuperScript IV reverse transcriptase (Thermo Fisher Scientific, Waltham, MA) and amplified by PCR using Q5 hot start PCR master mix (New England Biolabs, Ipswich, MA) and appropriate primers The 5′ terminal regions of the strains were amplified using the 5′ RACE System for Rapid Amplification of cDNA Ends (Thermo Fisher Scientific). The nucleotide sequences of the PCR products were determined by Sanger sequencing using the BigDye Terminator system (Thermo Fisher Scientific).

Parts of the viral cDNA regions corresponding to nucleotide positions 1–1624 (fragment A1), 1483–4062 (fragment A2), 3924–7848 (fragment B), 7729–10,141 (fragment C), and 10,028–10,748 (fragment D) of the ZIKV strains were amplified by RT-PCR with appropriate primers (Supplementary Table S2) as described above (Fig. 1). The 3′ terminal region of the viruses (terminal nucleotide positions 10,600–10,807; fragment E) was referred to as the sequence of PRVABC59 strain (accession No.: MH158237) and synthesized (Integrated DNA Technologies, Coralville, IA). cDNA fragments B, C, D, and E were concatenated into one fragment, BE, using joint PCR^37^. cDNA fragments were cloned into plasmid pMW119 for fragment A2 (Nippon Gene, Tokyo, Japan) and pMW119-CMVP for fragments A1 and BE, using an In-Fusion HD cloning kit (Takara Bio Inc., Shiga, Japan)^37,38^. The nucleotide sequences of the plasmid clones pZV-PRVABC59-A1, -A2, and -BE and pZV-NIID123-A1, -A2, and -BE were determined after amplification of the plasmids in *E. coli* NEB Stable (New England Biolabs). Fragments A1 with the CMV promoter sequence and A2 and BE with the HDV ribozyme sequence were PCR-amplified from the plasmid clones and then concatenated to form a full-length amplicon by joint PCR with appropriate primers (Supplementary Table S2). Vero cells were transfected with the full-length amplicon using X-tremeGENE HP DNA Transfection Reagent (Roche Diagnostics). At 6–7 days after transfection, culture supernatant was inoculated onto Vero cells and the cells were incubated at 37 °C for 4–7 days. The culture broth was used as the recombinant virus solution. The whole nucleotide sequences of the recovered recombinant viruses were determined as described above.

### Production of chimeric and point mutant ZIKV

Chimeric and point mutant ZIKV were generated by inverse PCR as described previously^52^, using appropriate primers (Supplementary Table S2). Briefly, the E regions of the PRVABC59 genome (nucleotide positions 978–1624 and 1483–2489) were amplified by RT-PCR as described above for the generation of the E protein chimera virus. The PRVABC59 E region cDNAs were ligated to PCR-amplified linearized pZV-NIID123 A1 and A2 of which the NIID123 E region was deleted, respectively. The resulting clones pZV-NIID123-A1-E^PRV^ and -A2-E^PRV^ were used to produce chimera virus rNIID123 E^PRV^ as described above.

For the generation of point mutant viruses, pZV-PRVABC59-A1 and pZV-NIID123-A1 were amplified by inverse PCR using primers with a nucleotide substitution at position 911 (positions 146 and 268 on prM and the polyprotein of ZIKV, respectively) (Supplementary Table S2), and the amplicons were circularized using the In-Fusion HD cloning kit. The resulting clones pZV-PRVABC59-A1-prM^L146F^ and pZV-NIID123-A1-prM^F146L^ were used to produce point mutant viruses rPRVABC59 prM^L146F^ and rNIID123 prM^F146L^, respectively, as described above.

### In vitro growth assay

Vero cells plated in a 6-well plate were infected with ZIKV at a multiplicity of infection (MOI) of 0.01 plaque forming units (pfu) or focus forming units (ffu) per cell. Small aliquots of the culture medium were collected periodically and the infectious titer in each aliquot was determined by the plaque assay or focus assay using Vero cells, as described previously^52,53^. For the focus assay, heat-inactivated anti-ZIKV (PRVABC59) serum from ZIKV-infected mice (described below) and horseradish peroxidase-conjugated anti-mouse IgG goat antibody (Thermo Fisher Scientific) were used as the first and second antibody, respectively. The focus was stained using a Peroxidase Stain DAB kit (Nacalai Tesque). Virus titers were compared using repeated-measures analysis of variance.

### Mouse experiment

Male 12–15-week-old IFNAR1-KO mice were subcutaneously inoculated with 1 × 10^4^ pfu or ffu of virus in 40 µl of MEM via the footpad. Blood was collected periodically from the tail vein until day 8 post infection. At 6 weeks post infection, all mice were euthanized and blood and testis samples were collected. Serum viral titers were measured by the plaque or focus assay. Viral RNA was extracted from serum using the High Pure Viral RNA kit. Each testis was homogenized and total RNA was extracted using TRIzol reagent (Thermo Fisher Scientific). The viral RNA copy number in each sample was determined by real-time RT-PCR with TaqMan probe, as described previously^34^. Viral RNA copy numbers were compared using the Kruskal–Wallis test followed by the Mann–Whitney test, in which the *p*-value was adjusted by the Bonferroni method.

### Evaluation of testis damage

The testes of ZIKV- or mock-inoculated mice were weighed and the mean and standard deviation (SD) of the testis weight in the mock-inoculated group were calculated. A weight below the mean minus 3SDs of testis weight of mock group was defined as atrophy. The ratios of atrophied to total testes in the groups were compared using Fisher’s exact test.

### Virus adaptation to Vero cells

Vero cells in a 25-cm^2^ flask were inoculated with rNIID123 E^PRV^ at an MOI of 0.001 ffu/cell and were incubated at 37 °C for 5 days. The culture supernatant was recovered and a 0.3-mL aliquot was used to reinoculate the Vero cells, which were incubated at 37 °C for 5 days. The passaging was repeated three times.

### Statistical analysis

Statistical analyses were conducted using EZR (Saitama Medical Center, Jichi Medical University, Saitama, Japan)^54^ and the data were visualized using Microsoft Excel.

## Supplementary Information

Supplementary Information.

## Data Availability

The authors will provide all the data and study materials upon request.
