# Supplementary material for: Leu-to-Phe substitution at prM146 decreases the growth ability of Zika virus and partially reduces its pathogenicity in mice
Source: Sci Rep. 2021 Oct 4;11:19635. doi: 10.1038/s41598-021-99086-2 (PMC8490429; doi:10.1038/s41598-021-99086-2)
Supplement: Supplementary file 1 — Supplementary Information. [file 41598_2021_99086_MOESM1_ESM.docx]

**Leu-to-Phe substitution at prM^146^ decreases the growth ability of Zika virus and partially reduces its pathogenicity in mice**

Takuya Inagaki^1, 2^, Satoshi Taniguchi^1^, Yasuhiro Kawai^3^, Takahiro Maeki^1^, Eri Nakayama^1^, Shigeru Tajima^1^, Haruko Takeyama^2^, Chang Kweng Lim^1^* & Masayuki Saijo^1^*

^1^Department of Virology I, National Institute of Infectious Diseases, Tokyo, Japan.

^2^Department of Life Science and Medical Bioscience, Waseda University, Tokyo, Japan

^3^Management Department of Biosafety and Laboratory Animal, Division of Biosafety Control and Research, National Institute of Infectious Diseases, Tokyo, Japan

Corresponding authors: msaijo@nih.go.jp (M. Saijo), ck@nih.go.jp (C. K. Lim)


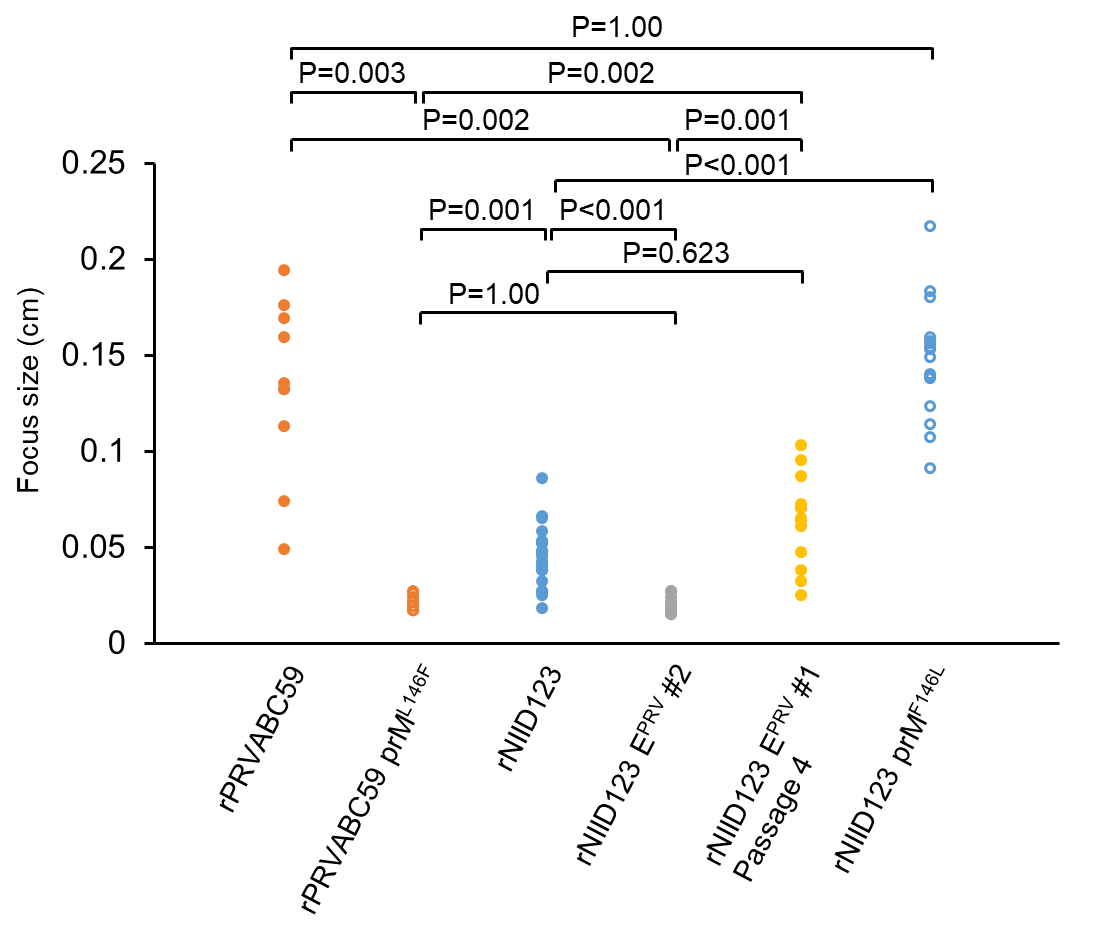
**Supplementary Figure S1.**

Data on focus size.

**Supplementary Table S1.** Amino acid differences on prM and E protein of ZIKV strains PRVABC59 and NIID123.

| Position  (polyprotein) | Protein-Position | paPRVABC59 | rPRVABC59 | paNIID123 | rNIID123 |
| --- | --- | --- | --- | --- | --- |
| 139 | prM-17 | Asn | Asn | Ser | Ser |
| 157 | prM-35 | Tyr | Tyr | His | His |
| 268 | prM-146 | Leu | Leu | Phe | Phe |
| 374 | E-84 | Lys | Lys | Arg | Arg |
| 550 | E-260 | Ser | Ser | Asn | Asn |
| 620 | E-330 | Val/Leu | Leu | Val | Val |

**Supplementary Table S2**. Primers used for the development of the reverse genetics system.

| Target or purpose of PCR primers | Orientation | sequence (5′ – 3′) |
| --- | --- | --- |
| Fragment A1 | forward | AGTTGTTGATCTGTGTGAATCAG |
|  | reverse | TCCTTGTGAACCAACCAGTGC |
| Fragment A2 |  |  |
| for PRVABC59 | forward | TAACGCCCAATTCACCGAGAG |
| for NIID123 | forward | TAACGCCCAATTCACCAAGAG |
|  | reverse | CTGCTCTCCACGCCACAAG |
| Fragment B | forward | GTTCTCATCAATGGTTTTGCTTTG |
|  | reverse | TTGCACTTCCTCGGGACACAG |
| Fragment C | forward | CCCTGGAGTTCTACTCCTAC |
|  | reverse | TGGTCGTTCTCCTCAATCCAC |
| Fragment D | forward | TGACTGGGTTCCAACTGGGAG |
|  | reverse | GCGTGGTGGAAACTCATGGAG |
| Cloning fragment A1 | forward | GTTGGTTCACAAGGAGGGGATCC |
|  | reverse | TGATTCACACAGATCAACAACTCGGTTCACTAAAC |
| Cloning fragment A2 | forward | TGGCGTGGAGAGCAGGGGGATCCTC |
|  | reverse | GTGAATTGGGCGTTACTAGCGGGTAC |
| Cloning fragment BE | forward | GAAATCCATGGGTCTGGGTCGGCATGGCATC |
|  | reverse | CAAAGCAAAACCATTGATGAGAACCTAGCGGGTAC |
| CMVp-fragment A1 | forward | CAGGGTTTTCCCAGTCACGA |
|  | reverse | TCCTTGTGAACCAACCAGTGC |
| Fragment BE-HDV ribozyme | forward | GTTCTCATCAATGGTTTTGCTTTG |
|  | reverse | TGTGTGGAATTGTGAGCGGAT |
| Combined whole-genome cDNA | forward | CGTTGTAAAACGACGGCCAG |
|  | reverse | TGACCATGATTACGCCAAGC |
| E region of pZV-PRVABC59-A1 | forward | ATCAGGTGCATAGGAGTCAGC |
|  | reverse | TCCTTGTGAACCAACCAGTGC |
| pZV-NIID123-A1 without E region | forward | GTTGGTTCACAAGGAGGGGATCC |
|  | reverse | TTGCTGACTCCTATGCACCTG |
| E region of pZV-PRVABC59-A2 | forward | TAACGCCCAATTCACCGAGAG |
|  | reverse | AGAGACGGCTGTGGATAAGAAG |
| pZV-NIID123-A2 without E region | forward | TCCACAGCCGTCTCTGCTG |
|  | reverse | GGCTCTCGGTGAATTGGGC |
| pZV-PRVABC59-A1-prM^L146F^ | forward | CGCTTGGCTTTTTGGAAGCTC |
|  | reverse | TCCAAAAAGCCAAGCGATG |
| pZV-NIID123-A1-prM^F146L^ | forward | CGCTTGGCTTTTGGGAAGCTC |
|  | reverse | TCCCAAAAGCCAAGCGATG |
